# Gonadotropins Activate Oncogenic Pathways to Enhance Proliferation in Normal Mouse Ovarian Surface Epithelium

**DOI:** 10.3390/ijms14034762

**Published:** 2013-02-28

**Authors:** Tyvette S. Hilliard, Dimple A. Modi, Joanna E. Burdette

**Affiliations:** Department of Medicinal Chemistry and Pharmacognosy, University of Illinois at Chicago, 900 S. Ashland Ave. Chicago, IL 60607, USA; E-Mails: thilli2@uic.edu (T.S.H.); dmodi2@uic.edu (D.A.M.)

**Keywords:** ovarian cancer, ovarian surface epithelium (OSE), gonadotropins, follicle stimulating hormone (FSH), luteinizing hormone (LH), normal 3D organ culture

## Abstract

Ovarian cancer is the most lethal gynecological malignancy affecting American women. The gonadotropins, follicle stimulating hormone (FSH) and luteinizing hormone (LH), have been implicated as growth factors in ovarian cancer. In the present study, pathways activated by FSH and LH in normal ovarian surface epithelium (OSE) grown in their microenvironment were investigated. Gonadotropins increased proliferation in both three-dimensional (3D) ovarian organ culture and in a two-dimensional (2D) normal mouse cell line. A mouse cancer pathway qPCR array using mRNA collected from 3D organ cultures identified Akt as a transcriptionally upregulated target following stimulation with FSH, LH and the combination of FSH and LH. Activation of additional pathways, such as Birc5, Cdk2, Cdk4, and Cdkn2a identified in the 3D organ cultures, were validated by western blot using the 2D cell line. Akt and epidermal growth factor receptor (EGFR) inhibitors blocked gonadotropin-induced cell proliferation in 3D organ and 2D cell culture. OSE isolated from 3D organ cultures stimulated with LH or hydrogen peroxide initiated growth in soft agar. Hydrogen peroxide stimulated colonies were further enhanced when supplemented with FSH. LH colony formation and FSH promotion were blocked by Akt and EGFR inhibitors. These data suggest that the gonadotropins stimulate some of the same proliferative pathways in normal OSE that are activated in ovarian cancers.

## 1. Introduction

Ovarian cancer is the most lethal gynecological malignancy and the fifth leading cause of cancer related death in American women [1]. The pathophysiology of ovarian cancer is poorly understood among major cancers due to limited understanding of etiological factors and mechanisms of cancer promotion. One of the major challenges in the field is lack of robust models that could establish early pathways responsible for disease initiation and promotion. Roughly 70% of women with ovarian cancer will die from the disease due to the lack of specific signs and symptoms, leading to late diagnosis. Ovarian cancer is categorized into four histotypes including serous, endometrioid, mucinous, and clear cell, with serous being the most deadly. Increasing evidence suggests that the hormonal microenvironment surrounding the ovary could potentially influence the development and promotion of ovarian cancer [2]. Until recently, ovarian cancers were believed to arise only from the ovarian surface epithelium (OSE). However, emerging evidence suggests that the fallopian tube epithelium (tubal epithelial cells or TEC) could also be a source of epithelial ovarian cancer (EOC) [3–5].

While the etiology of ovarian cancer is poorly understood, there are a number of proposed hypotheses, all of which incorporate ovulation as a component in ovarian cancer initiation and promotion. The gonadotropins, follicle stimulating hormone (FSH) and luteinizing hormone (LH), are implicated in the etiology of ovarian cancer primarily because the median age of onset of ovarian cancer typically intersects with menopause and physiologically elevated levels of FSH and LH [6,7]. High levels of gonadotropins during ovulation, loss of gonadal negative feedback during menopause, and premature ovarian failure are all thought to play a role in ovarian cancer [7,8]. The gonadotropin hypothesis is supported by the decreased risk of ovarian cancer from a reduction of exposure to FSH and LH by the use of oral contraceptives, multiparity, and breast-feeding [7], and an increased risk of the disease with early menarche, late menopause, nulliparity, and the use of fertility drugs [6,9]. However, the effects of gonadotropins on completely normal OSE cultured in the ovarian microenvironment are not fully characterized.

The gonadotropins, FSH and LH, are glycoprotein hormones synthesized in the anterior pituitary [7,10]. Both FSH and LH share a common α-subunit, but each has a distinctive β-subunit, which confers the specificity of the two hormones [11]. FSH is responsible for the maturation of immature follicles to Graafian follicles [12]. LH triggers ovulation and development of the corpus luteum. The effects of FSH and LH on granulosa and theca cells [13,14] have been published, but the role of these gonadotropins on normal OSE is less well characterized. FSH receptors (FSHR) and LH receptors (LHR) are expressed in normal OSE, normal fallopian tube epithelium, and ovarian tumor samples [7,15,16]. Several investigators have attempted to address the possible role of the gonadotropins in OSE using tumorigenic models as it pertains to EOC. *In vivo* studies demonstrate that FSH and LH increase OSE proliferation [17–19]. *In vitro* studies have reported that FSH or LH alone increases [6,16], decreases [20,21], or has no effect on cellular proliferation of OSE cells [7,22,23]. Tubal epithelial cells express FSH and LH receptors, but normal TECs do not proliferate in response to gonadotropins [8]. FSH and LH signal together *in vivo* in post-menopausal women and during ovulation these hormones are almost always found at the same time. However, very little has been published regarding the combined effects of gonadotropins on signaling in OSE or ovarian cancer.

The purpose of this study was to identify the pathways downstream of the gonadotropins in normal OSE and their contribution towards proliferation and oncogenesis. Many *in vitro* studies using SV40T immortalized OSE cells or *in vivo* studies using animal models have been reported to evaluate the role of FSH and LH, but these systems fail to separate ovulation and the effects of gonadotropins, do not use completely normal cells, or separate the cells from their microenvironment [7,10,24]. This study used two different model systems to evaluate the actions of gonadotropins on normal OSE function. A three-dimensional (3D) organ culture system was employed to study the role of gonadotropins in normal cells grown within their microenvironment in the absence of ovulation [25]. Simultaneously, the effects of gonadotropins on the OSE alone were studied using a normal mouse OSE cell line. FSH, LH and the combination of FSH and LH (FSH+LH) enhanced cellular proliferation by activating Akt signaling and upregulating pro-proliferative cyclin dependent kinases and anti-apoptotic Birc5.

## 2. Results and Discussion

### 2.1. Results

#### 2.1.1. Gonadotropins Enhance Proliferation of Normal OSE

**Figure 1:**
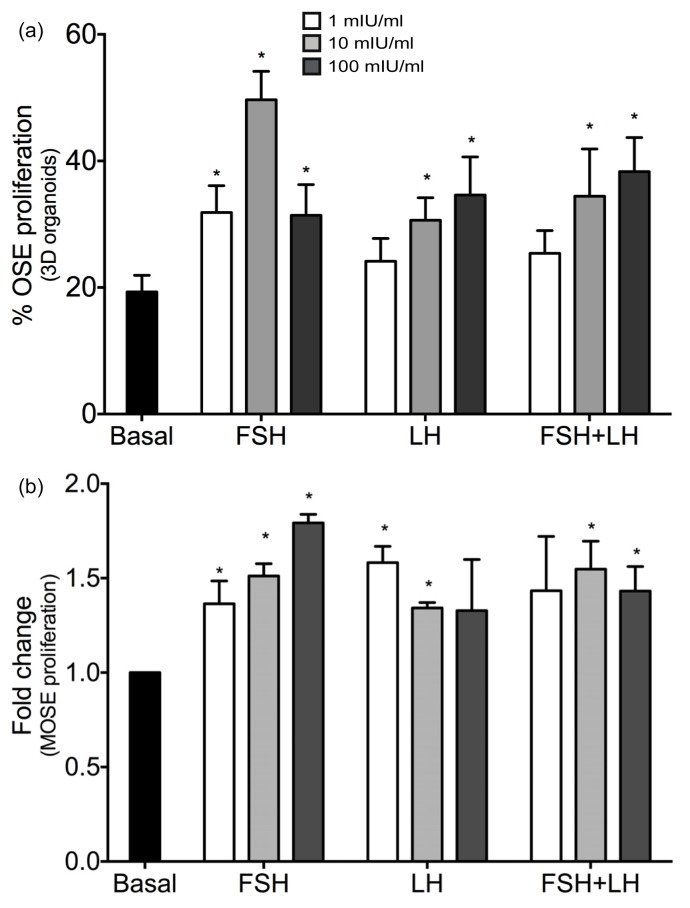
Gonadotropins increase ovarian surface epithelium (OSE) proliferation in 3D organoids and mouse ovarian surface epithelial (MOSE) cell line. (**a**) Using BrdU as a marker of DNA synthesis, the gonadotropins increased proliferation of OSE in organoids after 8 days. (**b**) Proliferation of MOSE cells in response to gonadotropin stimulation was measured by sulforhodamine B (SRB) assay after 8 days. * different than basal *p* < 0.05.

Gonadotropins have been reported to have widely variable growth stimulatory properties on OSE *in vitro*[26–28], but *in vivo* they seem to enhance proliferation [18,19]. Therefore, to further characterize the contribution of the gonadotropins to OSE proliferation, a 3D organ culture system that propagates normal OSE in an alginate hydrogel was employed. Ovarian organoids were cultured for 8 days with FSH, LH and the FSH+LH at a dose of 1, 10 or 100 mIU/mL, representing a range of physiologically relevant concentrations. To determine the percentage of proliferating cells, BrdU was incorporated into the culture media 24 h prior to fixation. FSH at all three doses in 3D significantly increased proliferation of OSE as compared to basal media, while LH and the combination of FSH and LH only significantly increased proliferation at 10 and 100 mIU/mL (Figure 1a). In order to compare the effects of FSH, LH and FSH+LH on OSE proliferation *in vitro*, 2D mouse ovarian surface epithelial cells (MOSE) were analyzed for proliferation after stimulation with gonadotropins for 8 days [29]. FSH at 1, 10 and 100 mIU/mL increased proliferation above basal levels, LH increased proliferation at 1 and 10 mIU/mL, and FSH+LH increased proliferation at 10 and 100 mIU/mL (Figure 1b).

#### 2.1.2. Gonadotropins Regulate Oncogenic Signal Transduction Pathways in Normal Mouse OSE Cultured in 3D

To investigate the signal transduction pathways altered in normal mouse OSE cultured in 3D that may be involved in the proliferative response observed following culture with the gonadotropins, organoids were cultured for 3 days in basal media followed by 24h incubation with 10 mIU/mL FSH, LH or FSH+LH. The organoids were incubated with collagenase to collect an enriched OSE cell preparation and the mRNA was subjected to a Cancer Pathway Finder qPCR array. The array identified several signal transduction pathways in OSE that were altered in response to FSH, LH and FSH+LH as compared to OSE from organoids cultured in basal media. The gonadotropins increased gene expression of some pro-proliferative factors, including Akt. Although both FSH and LH significantly amplified the Akt pathway, LH and FSH+LH amplified both Akt1 and Akt2 isoforms, while FSH only amplified the Akt2 isoform. The pro-proliferative epidermal growth factor receptor (EGFR) was upregulated more by FSH and FSH+LH than LH alone. FSH and FSH+LH treated OSE amplified cyclin-dependent kinase 2 (Cdk2) as well as Cdk4 mRNA expression when compared to basal cultured OSE. The anti-apoptotic factor, Birc5, was amplified more when treated with FSH and FSH+LH than LH alone (Table 1). The gonadotropins alone and combined also upregulated expression of angiopoietin 1, which is involved in vascularization, and reduced the expression of pro-apoptotic caspase 8. Expression of mRNA for cyclin-dependent kinase inhibitor 2A (Cdkn2a), which slows progression through the cell cycle, was increased in response to FSH, LH and FSH+LH.

#### 2.1.3. Gonadotropins Enhance Akt Expression in Normal OSE

**Figure 2:**
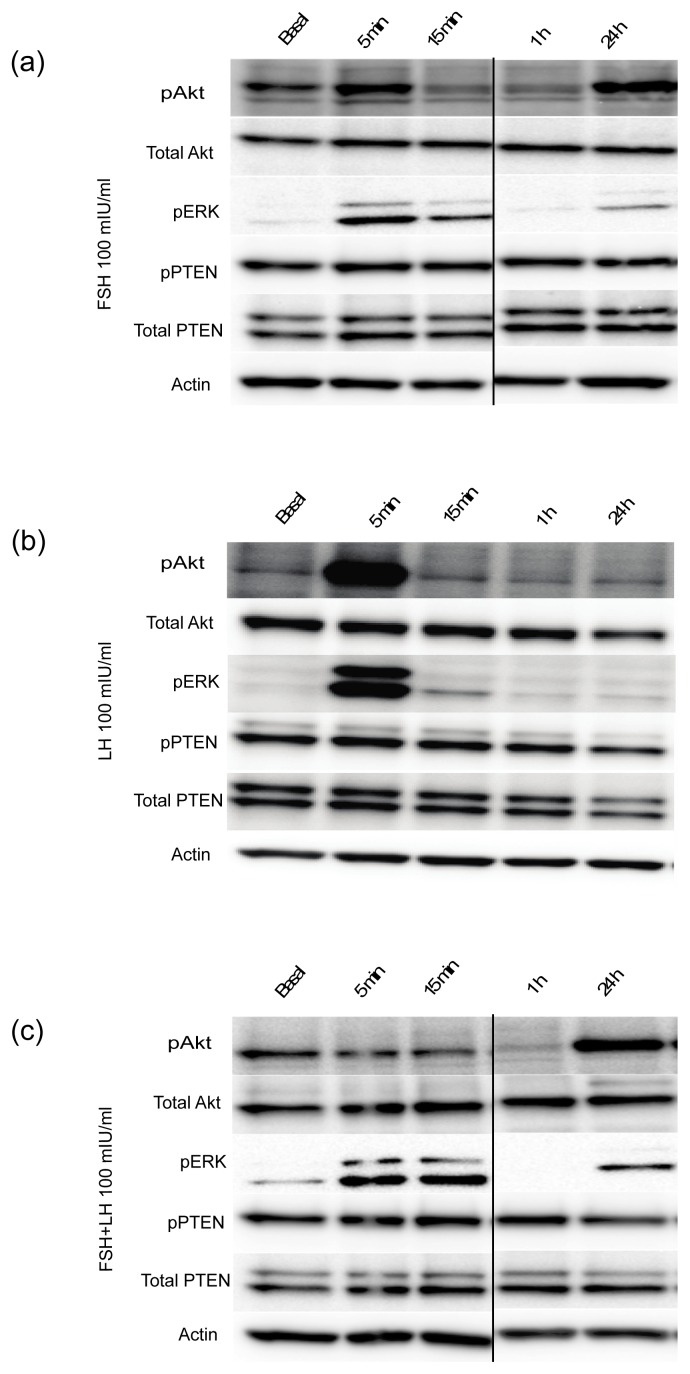
Gonadotropins upregulate pAkt and pERK expression in MOSE cells. Activated pAkt and pERK expression was observed in MOSE after treatment with 100 mIU/mL of (**a**) FSH; (**b**) LH or (**c**) FSH+LH. Activated p-PTEN and total PTEN levels were similarly probed. Protein was normalized to actin. All gels were run in 3 independent experiments with a representative image shown.

In order to evaluate the effects of gonadotropins on Akt expression in the OSE, MOSE cells were employed [29]. MOSE cells were treated with FSH, LH or FSH+LH at 100 mIU/mL each for 5 min, 15 min, 1 h, and 24 h. A significant increase in the expression of phosphorylated Akt (p-Akt) was noted after 5 minutes when stimulated with FSH or LH (Figure 2). FSH and FSH+LH also enhanced p-Akt expression after 24 h. Gonadotropin treatment did not affect the expression of total Akt in any of the treatment groups. The phosphatase PTEN inactivates Akt, and loss of homozygosity or mutation of this gene has been noted in ovarian cancer and endometrioid ovarian cancer, respectively [30,31]. Therefore, PTEN expression was analyzed to determine if its loss contributed to the activation of Akt. Levels of PTEN and p-PTEN were not altered upon stimulation with FSH, LH or FSH+LH (Figure 2). Because p-ERK expression has previously been noted downstream of FSH and LH in human immortalized OSE and in human ovarian cancer cells, the activation of p-ERK was investigated [26]. Expression of p-ERK in all three groups was elevated after 5 min, but did not persist after 24 h (Figure 2).

**Figure 3:**
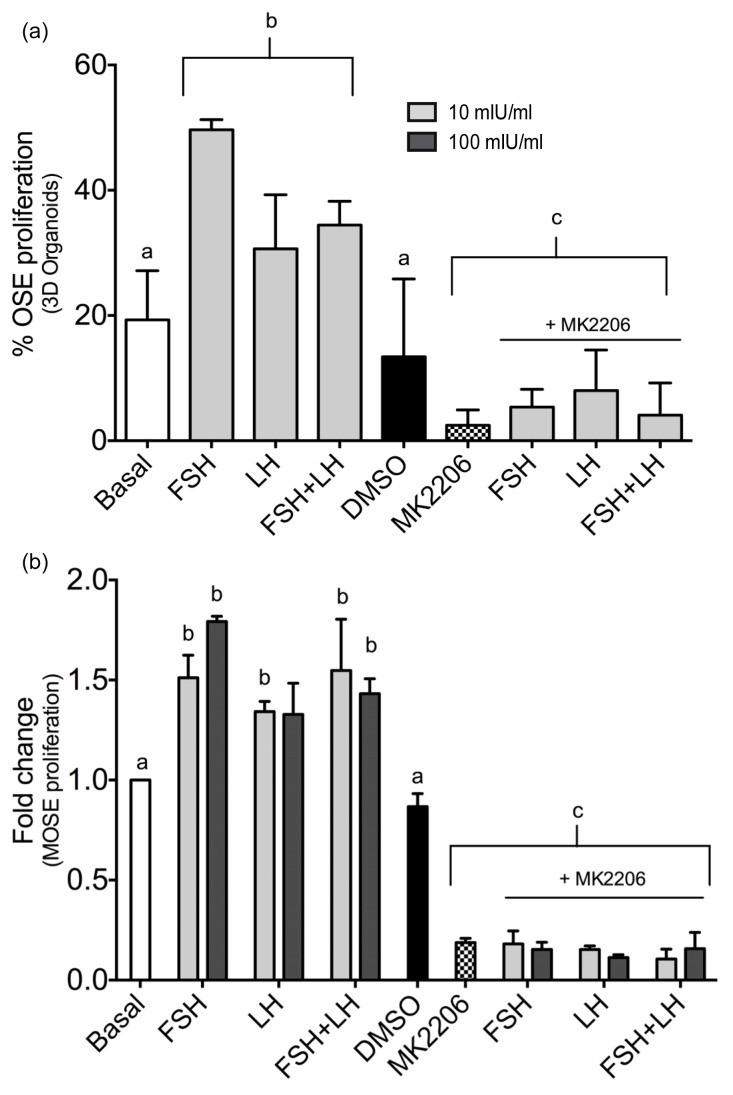
Inhibition of Akt signaling decreases gonadotropin regulated OSE proliferation. An Akt specific inhibitor MK-2206 significantly decreased gonadotropin induced OSE proliferation in (**a**) 3D organoids (10 μM) and (**b**) a MOSE cell line (2 μM). “a” is different than “b”; *p* < 0.05, “c” is different than groups treated with gonadotropins in absence of the inhibitor; *p* < 0.05.

The gonadotropins increased proliferation of the OSE in 3D organ culture (Figure 1). To determine if proliferation of the OSE induced by the gonadotropins could be blocked by inhibiting the Akt pathway, organoids or MOSE cells were cultured with gonadotropins in the presence of MK-2206, a chemical inhibitor of Akt phosphorylation. First, 5 μM MK-2206 was added to the MOSE culture media for 5 min in the presence of 100 mIU/mL of FSH or LH to validate that MK-2206 decreased p-Akt expression (Supplemental Figure 1a). Next, proliferation of OSE in 3D organ culture and 2D MOSE cells was monitored in the presence of gonadotropins combined with MK-2206. MK-2206 significantly decreased 3D OSE proliferation induced by FSH, LH or FSH+LH (Figure 3a). MOSE cells treated with the gonadotropins in the presence of MK-2206 exhibited a decreased rate of proliferation as compared to MOSE cells treated with the gonadotropins alone (Figure 3b). Inhibition of Akt also blocked basal levels of proliferation.

**Figure 4:**
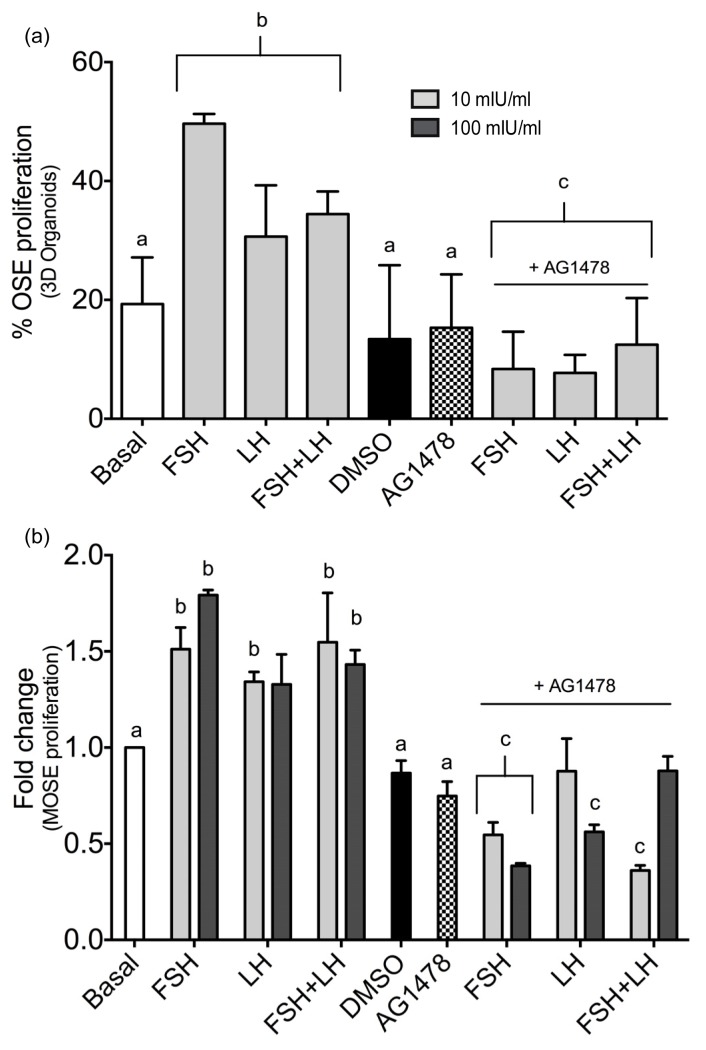
Inhibition of epidermal growth factor receptor (EGFR) signaling repressed OSE proliferation in (**a**) 3D and (**b**) MOSE cells. Proliferation of the OSE caused by the gonadotropins is blocked after 8 days of culture in the presence of EGFR inhibitor AG1478 (100 nM) in both 3D cultured organoids and 2D cultured MOSE cells. “a” is different than “b”; *p* < 0.05, “c” is different than groups treated with gonadotropins in absence of the inhibitor; *p* < 0.05.

The transcription pathway array indicated that EGFR mRNA was upregulated by gonadotropins, which has been observed in other studies using human ovarian cancer and normal OSE cells [26]. First, 100 nM AG1478 was added to the MOSE culture media for 5 min in the presence of 100 mIU/mL of FSH or LH to validate that AG1478 decreased p-ERK expression, which is downstream of EGFR (Supplemental Figure 1b). To determine if inhibition of EGFR signaling blocked OSE proliferation, organoids were cultured for 8 days with FSH, LH or FSH+LH alone or with AG1478. AG1478 decreased OSE proliferation stimulated by FSH, LH, or FSH+LH in 3D organ cultures (Figure 4a) and in MOSE cells treated with the gonadotropins (Figure 4b).

#### 2.1.4. Gonadotropins Increase Expression of Proliferative and Anti-Apoptotic Proteins in Normal OSE

**Figure 5:**
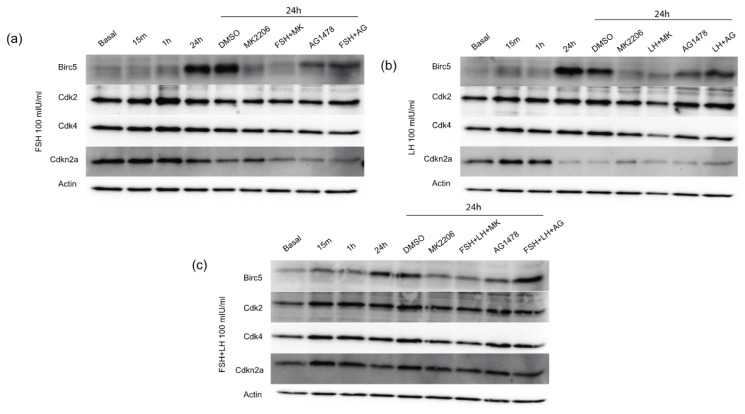
Gonadotropins regulate proliferative and anti-apoptotic signaling pathways in normal OSE downstream of Akt and EGFR. MOSE cells treated for 15 min, 1 h and 24 h with (**a**) FSH 100 mIU/mL; (**b**) LH 100 mIU/mL and (**c**) FSH+LH 100 mIU/mL was analyzed for Birc5, cdk2, cdk4, and Cdkn2a. MOSE cells were treated for 24 h with gonadotropins supplemented with the Akt inhibitor MK-2206 5 μM and AG1478 100 nM. Protein was normalized to actin. All gels were run in 3 independent experiments with a representative image shown.

To determine if the mRNA expression levels identified from the transcription array correlated with protein expression, western blot analyses were performed for Birc5, Cdk2, Cdk4 and Cdkn2a. MOSE cells were treated with serum-free basal media or the gonadotropins for 15 min, 1 h and 24 h. Birc5, also known as survivin, is an anti-apoptotic protein that could modulate the total number of cells as measured in the growth assays. Birc5 protein was upregulated by FSH, LH and FSH+LH at 24 h when compared with basal conditions (Figure 5). Incubation with the Akt inhibitor, MK-2206, was able to block Birc5 induction by FSH, LH and FSH+LH. The EGFR inhibitor, AG1478, did not antagonize gonadotropin induction of Birc5, indicating that FSH and LH regulate Birc5 expression through an Akt-dependent pathway. Despite the fact that DMSO enhanced basal levels of protein expression, the Akt and EGFR inhibitors were able to block DMSO and gonadotropin induced expression of proteins [32].

Cdk2 protein was increased by FSH, LH and FSH+LH at 24 h (Figure 5). Both the Akt inhibitor MK-2206 and the EGFR inhibitor AG1478 mitigated the induction of Cdk2 by FSH and FSH+LH. However, only MK-2206 reduced the expression of Cdk2 in LH treated MOSE cells. Cdk4 was induced by FSH, LH and FSH+LH (Figure 5). MK-2206 and AG1478 mitigated the gonadotropin induced Cdk4 expression suggesting that Cdk4 is located downstream of Akt and EGFR. Protein expression for cyclin-dependent kinase inhibitor 2A (Cdkn2a) was elevated at shorter time points, but was repressed after 24 h when treated with FSH and LH, despite the increase in mRNA expression identified by the transcription array. In the presence of FSH and LH alone, MK-2206 and AG1478 failed to relieve this repression of Cdkn2a at 24 h. FSH+LH failed to repress Cdkn2a expression at 24 h.

#### 2.1.5. Gonadotropins Enhance Soft Agar Colony Formation

**Figure 6:**
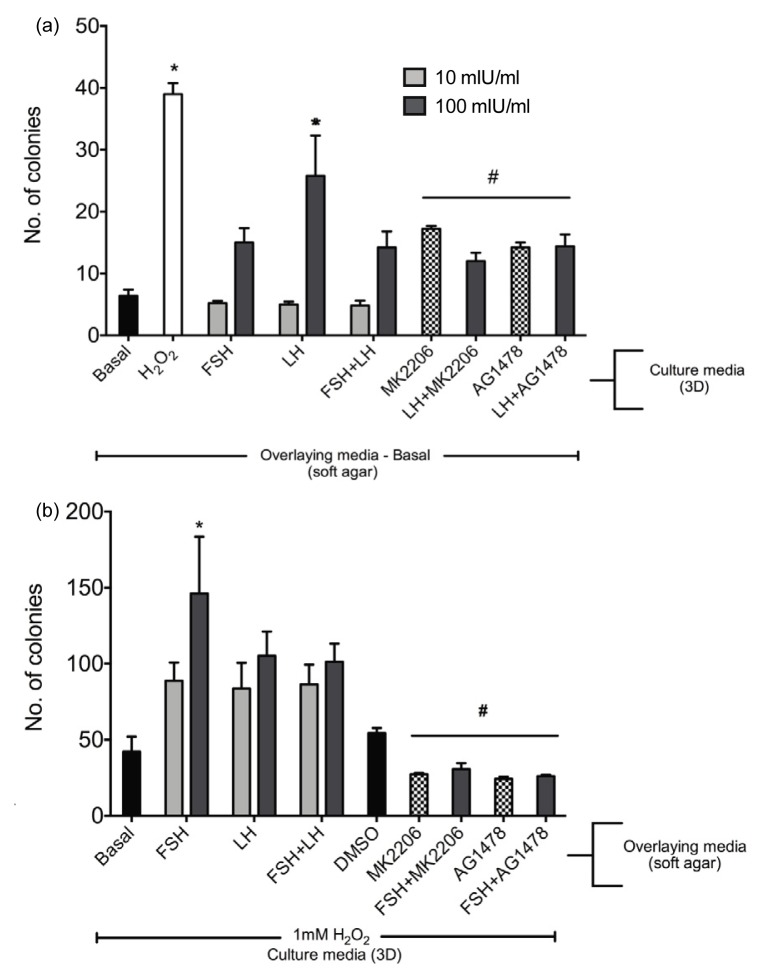
LH treated OSE increased anchorage independent growth. (**a**) OSE from 3D organoids cultured in 100 mIU/mL LH demonstrated an increase in colony formation when compared to basal cultured organoids that could be blocked with MK-2206 and AG1478 whereas (**b**) OSE cultured in 1 mM H_2_O_2_ and overlaid with FSH showed an increase in colony formation compared to OSE overlaid with basal medium that could be blocked with MK-2206 and AG1478. * different than basal; *p* < 0.05, “#” is different than *p < 0.05.

To determine if the gonadotropins enhanced growth of colonies in soft agar, OSE from 3D organoids were cultured in 10 and 100 mIU/mL FSH, LH or FSH+LH for 3 days. Following stimulation with gonadotropins, the OSE from the organoids was collected using collagenase as previously described [33]. Only OSE from the organoids cultured in 100 mIU/mL LH demonstrated a significant increase in the number of colonies compared to those cultured in basal medium (Figure 6a). FSH and the combination of FSH+LH did not enhance colony formation. In order to investigate if the LH-induced increase in colony formation was dependent on Akt and EGFR signaling pathways, MK-2206 and AG1478 were added to 3D organoids cultured with LH. Both the inhibitors significantly reduced the number of colonies compared to those cultured without the inhibitors. To determine if gonadotropins promoted colony formation, organoids were cultured in 1mM H_2_O_2_ for 3 days, a condition that previously supported soft agar colony formation [33] followed by addition of OSE to soft agar overlaid with FSH, LH or FSH+LH medium. H_2_O_2_-induced OSE colonies in soft agar overlaid with 100 mIU/mL FSH, showed a significant increase in number of colonies compared to those cultured in basal overlay media (Figure 6b). MK-2206 and AG1478 in presence of FSH in the media overlay significantly reduced the number of colonies formed compared to those formed by the OSE cultured with FSH alone.

### 2.2. Discussion

Ovarian cancer is commonly diagnosed in post-menopausal women, when the levels of FSH and LH are elevated [10]. The intersection between high levels of gonadotropins and the average age of incidence of ovarian cancer is the basis of a hypothesis suggesting a relationship between gonadotropins and an increased risk and incidence of ovarian cancer. Gonadotropins induced OSE proliferation in an *in vitro* 3D mouse model of primary OSE cells and in a normal mouse OSE cell line. Elevated levels of cell cycle regulatory and anti-apoptotic proteins that regulate proliferation were observed in MOSE cells treated with gonadotropins. LH stimulated colony formation of 3D cultured OSE in soft agar. FSH+LH was not able to completely mimic either hormone alone and reduced colony formation as compared to LH. Proliferation and colony formation could be blocked with both Akt and EGFR inhibitors indicating that these are important regulators of growth in normal OSE. FSH, LH, and the combination of FSH+LH induced Birc5, which was blocked when Akt was inhibited. FSH and FSH+LH induced Cdk2, which was reduced by Akt and EGFR inhibition. Overall these data indicate that the gonadotropins individually and in combination regulate proliferation, but the mechanisms of regulation by each hormone are different.

Proliferation of the OSE and its association with ovulation has been suggested to play a role in OSE transformation and cancer progression [18]. The results from this study are similar to *in vivo* findings of increased OSE proliferation in response to the gonadotropins in different animal models [17–19,34]. When comparing the 2D and 3D systems, OSE grown in 2D, stimulated with gonadotropins, began to proliferate much faster (day 2-data not shown) than in 3D (day 8). However, both 2D and 3D systems displayed enhanced proliferation after 8 days. The discrepancy between model systems implies that the architecture of the ovarian microenvironment likely impacts the proliferation of normal OSE. This report did not evaluate estrogen receptor (ER) signaling in the ovarian surface, which potentially could occur if the gonadotropins are stimulating the follicles to secrete estrogen in the organoid [6,35]. Further, the 2D proliferation assay and the CK8/BrdU labeled immunohistochemistry on 3D organ culture did not account for the ERα induced OSE signaling that could have occurred in addition to proliferation.

Investigating pathways involved in carcinogenesis allowed for the detection of a series of specific genes regulated in the OSE by FSH, LH and FSH+LH. Akt is a serine/threonine kinase that is activated in roughly 68% of ovarian cancers [30]. Akt1 is activated in ovarian cancer, Akt2 is overexpressed in primary tumors as well as human ovarian carcinoma cell lines [36–39], and Akt3 expression is elevated in 20% of ovarian cancers [40]. The qPCR array identified transcriptional upregulation of Akt1 and Akt2 by LH and FSH+LH, while FSH increased expression of only Akt2. The transcription array data indicated that total Akt expression was enhanced but it did not correlate with the 2D MOSE cell line data likely because the mRNA was from an enriched OSE preparation that contained some underlying stromal cells. In addition to stromal cells, the OSE preparation is likely to be contaminated with a small percentage of theca and granulosa cells that express gonadotropin and EGF receptors [14,41]. FSH and LH signaling via these receptors could account for the discrepancy in the fold change observed in Akt1 and Cdk4 in response to FSH, LH and FSH+LH. Immunohistochemistry indicated that the stroma had abundant expression of Akt after stimulation with gonadotropins (data not shown). However, the 2D cell line data does support that Akt is phosphorylated by the gonadotropins. The Cancer Genome Atlas Network did not identify mutation of Akt as the primary mechanism for its activation, suggesting that perhaps stimulation by gonadotropins, is one possible mechanism for the elevated p-Akt levels observed in ovarian cancer.

Epidermal growth factor receptor (EGFR) is a transmembrane glycoprotein that contains an external binding domain and an intracellular tyrosine kinase domain [42,43]. EGFR has been implicated in growth and progression of ovarian cancer, and may represent a prognostic indicator or potential therapeutic target [42]. EGFR overexpression has been shown to correlate with poor survival outcomes in women that have advanced ovarian cancer and have undergone cytoreductive surgery and combined therapy [43–45]. Inhibition of EGFR signaling by AG1478 in organ culture and MOSE cells significantly decreased OSE proliferation likely due to reduced expression of Cdk2 and Cdk4.

A soft agar assay demonstrated that post-menopausal concentrations of LH induced growth of OSE in soft agar. Initiation of anchorage–independent growth was recently demonstrated in non-tumorigenic ovarian epithelial cells that overexpressed β-hCG, which is a ligand that also activates LHR [46]. Overexpression of the hormone specific β-subunit of hCG induced cell cycle progression through elevated cyclin D1, Cdk2 and Cdk4 expression, similar to the results reported by this study [46]. Interestingly, when LH was combined with FSH it was no longer capable of inducing transformation, suggesting that FSH may somehow block LH induced transformation. Furthermore, when colony formation was initiated by exposure to oxidative stress, LH did not further enhance colony growth. However, FSH increased the formation of colonies that were derived from oxidative stress. We have recently demonstrated that oxidative stress transforms OSE by activating Akt, damaging DNA, and stimulating secretion of an ovarian stromal factor [33]. An interesting future direction will be to determine if FSH functions similarly to the stromal factor to enhance colony formation downstream of DNA damage and Akt activation.

While the gonadotropins are typically studied as individual hormones, the current study attempted to monitor OSE proliferation when exposed to both hormones simultaneously. In postmenopausal women as well as during ovulation, FSH and LH are almost always circulating at the same time. Since the circulating levels of FSH and LH fluctuate periodically during the month, it is challenging to recapitulate the exact ratio of FSH and LH experimentally. Western blot analysis revealed that FSH+LH regulated the expression of proliferative proteins differently than the individual treatments of FSH or LH. FSH, LH, and FSH+LH were all able to stimulate the anti-apoptotic protein Birc5, which possibly accounts for the overall increase in survival at day 8. Birc5 was blocked by MK-2206, but not AG1478, indicating that Birc5 is more heavily regulated by Akt compared to EGFR. Intriguingly, average proliferation of OSE cultured in both 2D and 3D was lower when the gonadotropins were given simultaneously as compared to FSH alone. Furthermore, the combination of FSH and LH led to growth of fewer colonies in soft agar than LH alone. This may be reflective of the observation that the combination of gonadotropins reduced p-Akt expression after 5 minutes as compared to FSH alone. Interestingly, FSH and LH alone repressed the cell cycle inhibitor Cdkn2a expression at 24 h, which was not seen with the combination of FSH+LH. Taken together, these findings support the hypothesis that gonadotropins affect specific oncogenic signaling pathways that enhance proliferation in normal mouse OSE.

## 3. Experimental Section

### 3.1. Animals

Day 16 female CD1 mice were used for organ culture experiments. Animals were not primed with eCG. All mice were acquired through in-house breeding, and all breeders were purchased from Harlan (Indianapolis, IN, USA). Animals were treated in accordance with the National Institutes of Health Guide for the Care and Use of Laboratory Animals and the established Institutional Animal Care and Use protocol at the University of Illinois at Chicago. Animals were housed in a temperature and light-controlled environment (12 h light:12 h darkness) and were provided food and water *ad libitum*.

### 3.2. Organ Culture

Ovaries from pre-ovulatory day 16 mice were dissected as previously described [25,47]. Briefly, ovaries were dissected in dissection media composed of Liebovitz media with L-glutamine, 100 U penicillin (Gibco), and 100 μg/mL streptomycin. The bursa was removed with forceps and ovaries were cut with a scalpel into two or four pieces, termed organoids. Each organoid was placed into a 0.5% *w*/*v* alginate/PBS droplet formed on mesh fiber. The alginate-encapsulated organoid was placed into 50 mM CaCl_2_ for 2 min to cross-link the alginate, forming a gel around the organoid. The organoid was then placed in growth media that consisted of alpha-MEM (Invitrogen), 100 U penicillin (Gibco), and 100 μg/mL streptomycin. To study the effects of the gonadotropins, 1, 10 or 100 mIU/mL of human FSH (Sigma-Aldrich, St. Louis, MO, USA), human LH (Sigma-Aldrich) or FSH+LH, each at the same concentration, was added to the basal culture media. Bromodeoxyuridine (BrdU; 10 μM) was added into culture media 24 h prior to fixation to label proliferating cells.

### 3.3. Cell Culture

Mouse ovarian surface epithelium cells (MOSE; Barbara Vanderhyden, University of Ottawa, Ottawa, ON Canada) were grown in alpha-MEM (Mediatech, INC, Manassas, VA, USA), 1% l-glutamine, 10% fetal bovine serum (FBS; Gibco), epidermal growth factor (2 μg/mL; Roche, Indianapolis, IN, USA), insulin/transferrin/selenium (insulin 5 μg/mL; transferrin 5 μg/mL; selenite 5 ng/mL; Roche), gentamycin (1 μg/mL; Mediatech), and penicillin-streptomycin (0.5 μL/mL). MOSE cells from passage numbers 7–13 were used for all experiments in this paper. MK-2206 (Selleck Bio, Houston, TX, USA) or AG1478 (Cell Signaling, Cambridge, MA, USA) were added to cultures to inhibit Akt or EGFR activation, respectively.

### 3.4. Immunohistochemistry

Organoids were removed from culture media and fixed as previously described [25] using reagents from Vector Labs Inc. (Burlingame, CA, USA) unless otherwise noted. Immunohistochemistry was performed as previously described [8,18,25,47,48]. Section thickness for the BrdU/CK8-labeled tissue was 5 microns. Sections were mounted on Superfrost^®^Plus Microscope slides (Fisher Scientific, Hampton, NH, USA). Briefly, heat-induced antigen retrieval was performed using 10 mM sodium citrate. Tissues being stained for BrdU were treated with 4 M hydrochloric acid for 10 min followed by 10 min incubation with 0.1 M sodium tetraborate. Tissues were blocked with 3% H_2_O_2_, avidin and biotin for 15 min each. Control slides received serum block instead of primary antibody. The primary anti-bodies against BrdU (rat, 1:200; Abcam, Cambridge, MA, USA) and cytokeratin 8 (CK8) (TROMA-1 antibody, rat, 1:100; Developmental Studies Hybridoma Bank, Iowa City, IA, USA) were incubated overnight at 4 °C. Slides were washed and incubated with biotinylated secondary antibody in 3%-BSA-TBS. Following three washes in TBS-Tween, slides were incubated for 30 min in avidin/biotin complex (ABC). The antigen-antibody-HRP complex was visualized using diaminobenzidine reagent for 3–5 min and counterstained with hematoxylin. All conditions had a minimum of 5 organoids and the experiment was performed in triplicates.

### 3.5. RNA Isolation and RT-PCR

After culture of organoids in basal media for 3 days followed by 24 h treatment with FSH, LH, or FSH+LH, OSE was collected by collagenase digestion as described previously [47]. RNA was isolated using a Qiagen Qiashredder column and Qiagen RNeasy (Valencia, CA, USA) kit according to manufacturer’s protocol. cDNA was synthesized using SABiosciences RT^2^ First Strand Kit and was added to a RT^2^ Profiler PCR Mouse Cancer PathwayFinder Array (SABiosciences, Frederick, MD, USA). Cycling conditions for reactions were 95 °C for 10 min; 40 cycles of 95 °C for 15 s, and 60 °C for 1 min. Gene expression was calculated using ΔΔCt method and expressed as fold change compared to basal conditions from duplicate arrays.

### 3.6. Cell Viability Assay

MOSE cells were seeded into 96 well plates at 1 × 10^4^ cells/100 μL of media with serum. The next day cells were treated with FSH, LH, or a combination of FSH and LH at 1, 10 and 100 mIU/mL. The cells were allowed to grow for 8 days. Proliferation was measured using sulforhodamine B (SRB) colorimetric assay as described previously [49]. Media was aspirated and cellular proteins were fixed to the plate with 20% trichloroacetic acid for 1 h. Cells were washed with water and stained for 30 min with sulforhodamine B. Excess dye was washed with 1% (*v*/*v*) acetic acid. The protein-bound dye was re-suspended in 10 mM Tris buffer. Spectrophotometric analysis was completed using a Biotek Synergy 2 multi-mode microplate reader (Biotek, Winooski, VT, USA). All conditions were tested in three replicates in triplicate experiments.

### 3.7. Soft Agar Transformation Assay

OSE were collected from organoids cultured for 3 days in media containing FSH, LH, or FSH+LH, followed by analysis of anchorage-independent growth as measured by growth in soft agar. The base layer of the agar consisted of DMEM (Gibco), and 0.5% agarose (Sigma). The top layer consisted of DMEM, 0.35% agarose and 15 × 10^3^ cells/well in a 24 well plate. The agar was overlaid with DMEM, 4% FBS, and penicillin-streptomycin. After 14 days, colonies were imaged on a Nikon Eclipse TS100 using a DS-Ri1 digital camera and counted using NIS Elements software. All conditions were tested in three replicates in triplicate experiments.

### 3.8. Western Blotting

MOSE cells were cultured in basal media, serum starved overnight and treated with the gonadotropins for specified periods of time. MOSE cells and organoids were washed with ice cold PBS and lysed in RIPA buffer (25 mM Tris-HCl pH 7.6, 150 mM NaCl, 1% (*v*/*v*) Triton-X, 1% (*v*/*v*) sodium deoxycholate, 0.1% SDS), 1X Complete Mini Protease Inhibitor Cocktail tablets (Roche, Indianapolis, IN, USA), and 1X Phosphatase Inhibitor Cocktail III (Sigma). The bicinchonic acid assay (Pierce, Rockford, IL, USA) was used to determine protein concentration. Cell lysate (30 μg) was run on a 10% and 15% SDS-PAGE gel under reducing conditions and transferred to a nitrocellulose membrane. Membranes were blocked for 1 h at RT in 5% bovine serum albumin (for p-Akt, Akt (pan), PTEN, p-PTEN, p-ERK, Birc5, Cdk2) or 5% nonfat dry milk (for Actin, Cdk4, Cdkn2a) in Tris-buffered saline with 0.1% Tween-20 (TBS-T). Primary antibodies against phospho-Akt, Akt (pan), PTEN, p-PTEN, p-ERK (rabbit, 1:000 dilution; Cell Signaling Technology), Birc5, Cdk2 (rabbit, 1:500 dilution; Cell Signaling Technology) and Cdk4 (mouse, 1:1000 dilution; Cell Signaling Technology) were incubated on blots overnight at 4 °C. After washing in TBS-Tween, membranes were incubated with goat anti-rabbit or goat anti-mouse horseradish peroxidase secondary antibody (Cell Signaling) at 1:1000 in blocking buffer for 1 h. Membranes were washed and visualized using SuperSignal West Femto substrate (Thermo Scientific, Rockford, IL, USA) on a Protein Simple gel documentation system (Santa Clara, CA, USA). All gels were run in three individual experiments with the representative image shown.

### 3.9. Imaging and Counts

Immunohistochemistry images were captured with a Nikon E600 microscope using a DS-Ri1 digital camera and NIS Elements software (Nikon Instruments, Melville, NY, USA). Using ImageJ software (National Institutes of Health, Bethesda, MD, USA), the number of CK8-positive OSE cells that were also positive for BrdU were counted and expressed as percentage of total CK8-positive OSE cells. Soft agar colony images were taken on a Nikon Eclipse TS100 using a DS-Ri1 digital camera. NIS elements software was used to determine the number of colonies.

### 3.10. Statistical Analysis

Values were expressed as the mean ± S.E.M. Dunnett’s multiple comparison test to assess differences between control groups and experimental groups. A Student’s *t*-test was used for comparison between two groups. *p* < 0.05 was considered statistically significant.

## 4. Conclusions

Our study supports that gonadotropins induce proliferation in normal OSE cells of ovarian organ culture as well as MOSE cells. Gonadotropins regulate proliferation, cell cycle progression, apoptosis, and growth in soft agar. An improved understanding of molecular signaling mechanisms in normal OSE may help to identify novel targeted therapeutic approaches to slowing the growth of ovarian cancers derived from this cell type.

## Supplementary materials

Figure S1 Gonadotropin induced (**a**) p-Akt protein expression in MOSE cells was blocked in presence of an Akt inhibitor, MK-2206 at 5 min while (**b**) p-Erk protein expression was suppressed in presence of EGFR inhibitor, AG1478 at 5 min.

**Table S1:** Modified version of table 1. List of publications corresponding to gonadotropin-induced upregulation of specific genes listed in the table.

Gene	Effect of FSH and/or LH	Ovarian cancer cell line	Reference
**Proliferation/Cell cycle control**			

A	FSH induces pAkt expression	SKOV3 cells	[ 15]
	
kt	FSH and LH induce pAkt expression	IOSE80-PC	[ 26]
1			
A			
kt			
2			

Cdk2	Overexpression of β-hCG induces elevated expression of Cdk2 and Cdk4 proteins	T29 & T80 cells	[ 46]

Cdk4			

Cdkn2a			

Chek 2			

Cyclin D1	FSH increases expression of Cyclin D1	SKOV3 cells	[ 50]

EGFR	FSH and LH upregulate EGFR expression	IOSE80-PC and OVCAR3 cells	[ 26]

Mdm2			

Myc	FSH induced expression of c-Myc	OVCAR3 cells	[ 51]

Trp53			

**Angiogenesis/Metastasis/Adhesion**			

Angpt1			

Mcam			

c-Met			

S100a4			

Vegfc	FSH and LH stimulate VEGF expression	AO cells	[ 52]

	FSH stimulates VEGF expression	SKOV3 cells	[ 50]

**Apoptosis**			

Birc5	LH upregulates survivin expression	SKOV3 and MCV152 cells	[ 53]

	FSH upregulates survivin expression	SKOV3 cells	[ 50]

Casp8			

## Figures and Tables

**Table 1:** Oncogenic pathways modulated by gonadotropins. The OSE from follicle stimulating hormone (FSH), luteinizing hormone (LH) or FSH+LH treated organoids was removed using collagenase and mRNA was isolated. cDNA was reverse transcribed and added to a RT^2^ Profiler PCR Mouse Cancer Pathway Finder Array. Numbers represent fold change compared to basal cultured organoids of pathways regulated by the gonadotropins.

Proliferation/Cell Cycle Control			

Gene	FSH	LH	FSH+LH
Akt1	3.5	61	55
Akt2	58	58	37
Cdk2	3.7	1.0	2.6
Cdk4	22	0.9	175
Cdkn2a	38	25	17
Chek 2	1.6	0.7	1.0
Cyclin D1	1.4	0.6	1.3
EGFR	5.8	1.8	5.3
Mdm2	3.0	1.0	2.1
Myc	1.7	0.7	1.4
Trp53	2.5	1.0	2.0

**Angiogenesis/Metastasis/Adhesion**			

Angpt1	32	16	4.9
Mcam	3.0	1.2	2.0
c-Met	2.1	0.70	2.4
S100a4	3.2	0.6	2.2
Vegfc	3.5	1.1	2.4

**Apoptosis**			

Birc5	2.2	1.2	2.6
Casp8	0.92	0.1	0.45
