# Supplementary figures and images for: Gonadotropins Activate Oncogenic Pathways to Enhance Proliferation in Normal Mouse Ovarian Surface Epithelium
Source: Int J Mol Sci. 2013 Feb 28;14(3):4762–82. doi: 10.3390/ijms14034762 (PMC3634497; doi:10.3390/ijms14034762)

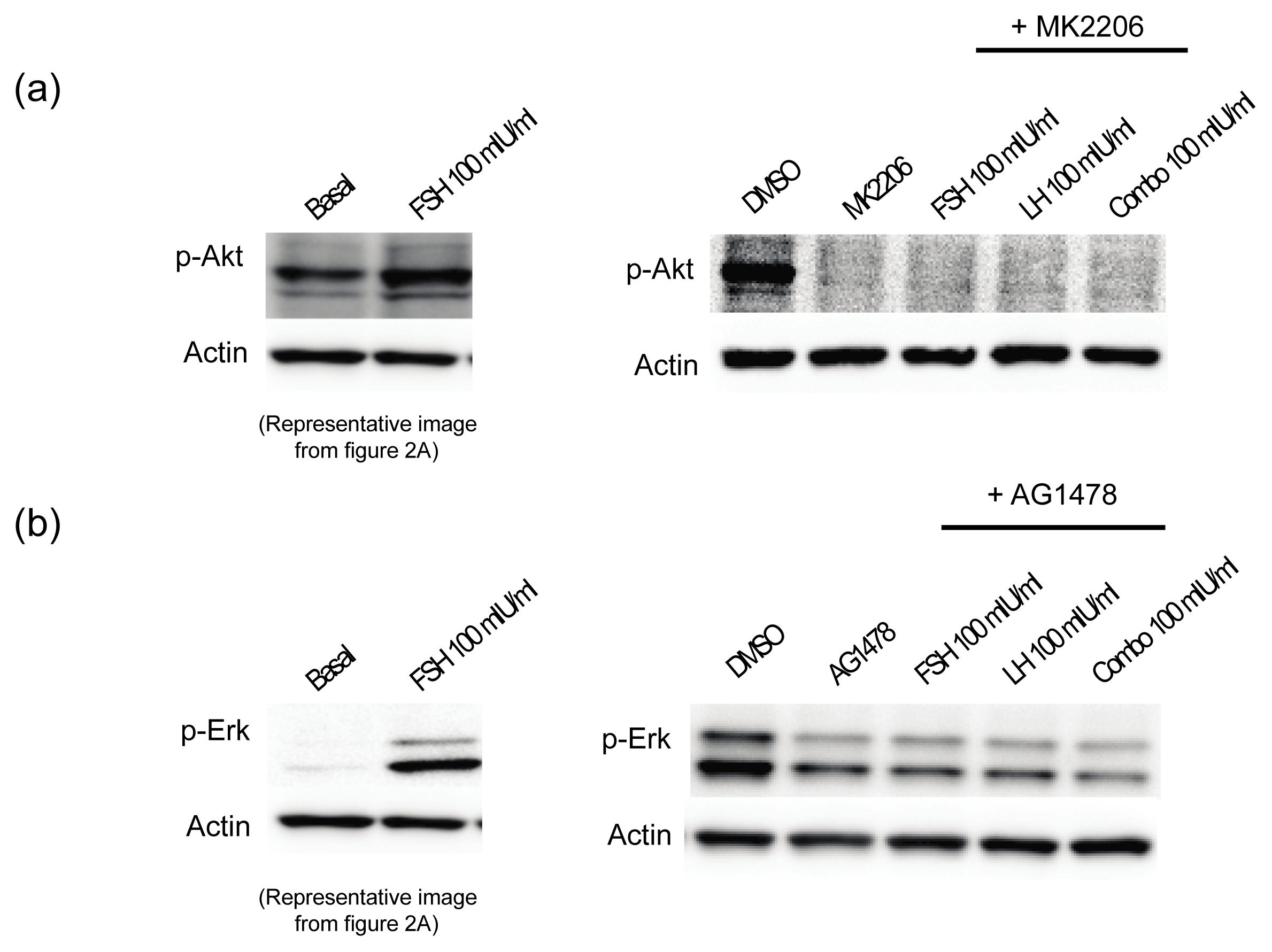

Supplement: Figure S1 — Gonadotropin induced (a) p-Akt protein expression in MOSE cells was blocked in presence of an Akt inhibitor, MK-2206 at 5 min while (b) p-Erk protein expression was suppressed in presence of EGFR inhibitor, AG1478 at 5 min. [file ijms-14-04762s1.tif]
